# A Computational Investigation of Occlusive Arterial Thrombosis

**DOI:** 10.21203/rs.3.rs-3011328/v1

**Published:** 2023-06-05

**Authors:** Jian Du, Aaron Fogelson

**Affiliations:** 1 Department of Mathematical Sciences, Florida Institute of Technology, 150 W. University BLVD, Melbourne, 32901, Florida, USA.; 2 Departments of Mathematics and Biomedical Engineering, University of Utah, 155 South 1400 East, Salt Lake City, 84112, Utah, USA.

**Keywords:** continuum model, platelet aggregation, occlusive thrombi, high shear flow

## Abstract

The generation of occlusive thrombi in stenotic arteries involves the rapid deposition of millions of circulating platelets under high shear flow. The process is mediated by the formation of molecular bonds of several distinct types between platelets; the bonds capture the moving platelets and stabilize the growing thrombi under flow. We investigated the mechanisms behind occlusive thrombosis in arteries with a two-phase continuum model. The model explicitly tracks the formation and rupture of the two types of interplatelet bonds, the rates of which are coupled with the local flow conditions. The motion of platelets in the thrombi results from competition between the viscoelastic forces generated by the interplatelet bonds and the fluid drag. Our simulation results indicate that stable occlusive thrombi form only under specific combinations for the ranges of model parameters such as rates of bond formation and rupture, platelet activation time, and number of bonds required for platelet attachment.

## 1 Introduction

Platelet processes, specifically platelet adhesion to the vessel wall and platelet aggregation with other platelets, play dominant roles in the formation of an intravascular blood clot (thrombus) in a stenotic artery. In the face of high shear conditions in constricted arteries, the platelets’ ability to form an aggregate and for that aggregate to remain intact is determined by interactions between fluid dynamics and multiple platelet attachment processes. The latter are mediated by protein receptors on platelet surfaces and bridging proteins in the plasma. As platelets aggregate during thrombus formation, they create a porous network with voids and channels, the spatial extent and porosity of which changes with time as additional platelets are recruited to the thrombus and individual platelets or pieces of the thrombus embolize (break away). With the changes in thrombus’ size and porosity, the drag forces exerted on it by the motion of fluid over and through it change as well and thereby dynamically alter the challenge platelet-platelet bonds collectively face in keeping the thrombus intact.

Platelet-platelet binding depends on interactions between the plasma proteins von Willebrand factor (vWF) and fibrinogen and the receptors GPIb*α* and integrin *α*_IIb_*β*_3_, respectively, on platelet surfaces [1]. There are approximately 25,000 GPIb*α* receptors and 50,000–80,000 *α*_IIb_*β*_3_ receptors on a typical platelet. The two types of bonds are distinguished (i) by whether the platelet is required to have been previously activated and (ii) by having substantially different kinetics. Fibrinogen binding to a platelet’s *α*_IIb_*β*_3_ receptors requires that platelet’s prior activation while vWF-GPIb*α* binding does not. Formation and breaking of *α*_IIb_*β*_3_-mediated bonds is slow compared to that of GPIb*α*-mediated bonds [2–5]. The breaking rates of both types of bond change with the load on them; fibrinogen-*α*_IIb_*β*_3_ are “slip” bonds [6] whose breaking rate increases with load, while vWF-GPIb*α* show “catch” and “slip” behaviors depending on the force sustained by the bonds [5], with the breaking rate actually decreasing under low-moderate loads and then increasing under larger one. (The vWF-GPIb*α* bond is also referred to as a “flex bond” [7]) The different bond kinetics influence the bonds’ effectiveness under different flow regimes and during different phases of the aggregate formation. Fibrinogen is present at high concentration in the blood plasma [8] while vWF has a much lower concentration. The ability of vWF to mediate platelet-platelet cohesion is believed to increase under high shear conditions where vWF molecules are found in a stretched configuration in which many otherwise hidden binding sites for platelet GPIb*α* are exposed [9].

Platelets and arteries have very different spatial scales, micron and millimeter, respectively. Dynamic interactions at the platelet scale are critical to events at the vessel scale. Our two-phase platelet aggregation model [10, 11], which is the basis of the work reported here, is at the macroscopic vessel scale. However, we derived it from an underlying *two-scale* model in which both the vessel scale and the much smaller platelet scale are treated. The two-scale model explicitly tracks the distribution of protein-mediated bonds between platelets based on the bonds location, orientation, and length, and uses the bond distribution function to calculate the stresses that the bonds exert when stretched. Because the model in this paper comes from a two-scale model, there is a close relationship between parameters of the macroscale model and cell- and molecular-level measurements available in the literature. Our model has the form of a two-phase mixture model in which the fluid and the bound platelets can move at different velocities. The use of two phases, with the possibility of relative motion between them, is critical for the formation of dense thrombi on physiological timescales [10, 11], features that our earlier single-phase models [12–14] were unable to fully capture.

Other continuum models of platelet aggregation treat the edge of the growing thrombus as a moving boundary and ignore the mechanics of the thrombus itself [15–17], or as an irreversibly growing impermeable elastic solid [18], or as a single phase viscoelastic material [19]. Recently, phase-field models were used to study the mechanics of a preformed thrombus [20, 21]. These models handle the thrombus’ mechanical properties using an energy functional without explicit reference to bond formation and breaking. The effect of vWF elongation is included indirectly in a two-phase continuum model of thrombus formation [22] by increasing the rate of platelet binding to and decreasing the rate of platelet detaching from the thrombus where flow conditions suggest that a large portion of vWF molecules would likely be in a stretched configuration. The binding and unbinding rates of platelets in that study are described using empirical functions.

In this paper, we describe extensions to our two-phase model [10, 11] to incorporate new experimental data about the formation of vWF-GPIb*α* bonds [23] and we apply the model to look at the formation of occlusive thrombi in stenotic vessels under pathologically high-shear flow conditions. In this setting, competition between drag on the thrombus and the formation of bonds able to resist the drag force is key to whether an occlusive thrombus develops at all, and whether, if one does develop, it is stable and long-lasting. To determine the drag forces, we use clot permeability data measured in the Ku lab [11] from thrombi formed under arterial flow. The model includes the dynamics and mechanics of both vWF-GPIb*α* bonds and fibrinogen-*α*_IIb_*β*_3_ bonds. This allows us to explore the impact on thrombus development and stability of the tension-dependent binding kinetics for vWF-GPIb*α* bonds recently suggested in [23] compared to the high constant rate of binding used in [4]. We also investigate the impact of variations on the Bell model breaking rate [24] for vWF-GPIb*α* bonds under high loads; studies of bond rupture in the literature are generally limited to loads of 100 pN or less [5, 6], and we consider how thrombus development might change if the breaking rate grew less rapidly for loads beyond 100 pN.

In the next section, we present our two-phase model of platelet thrombus formation and describe the changes to it made for the current studies. Then, we present and discuss the results of computational experiments looking at platelet deposition in a stenotic channel with a highest shear rate of 5,000/s. Finally, we draw some conclusions from these experiments.

## 2 Model

### 2.1 Two-phase Continuum Model for Platelet Aggregation

We next briefly describe our two-phase continuum model of platelet aggregation. More details about the model and its derivation can be found in [10, 11].

#### 2.1.1 Evolution Equations for Different Platelet Populations and Chemical Agonist

The model involves four platelet populations: mobile unactivated; mobile activated; bound unactivated; and bound activated, whose respective number densities are denoted by *ϕ*_u_, *ϕ*_a_, *ϕ*_bu_, and *ϕ*_ba_. The model involves one platelet activating chemical, which we think of as ADP, with concentration *c*. These variables change at rates described by the equations

(1) $${\left(\phi_{\text{u}}\right)}_{t}+\nabla \cdot \left(u_{\text{f}}\phi_{\text{u}}\right)=\nabla \cdot \left(D\nabla \phi_{\text{u}}\right)+f_{\text{bu}}^{\text{u}}-f_{\text{u}}^{\text{bu}}-f_{\text{u}}^{\text{a}}$$

(2) $${\left(\phi_{\text{a}}\right)}_{t}+\nabla \cdot \left(u_{\text{f}}\phi_{\text{a}}\right)=\nabla \cdot \left(D\nabla \phi_{\text{a}}\right)+f_{\text{u}}^{\text{a}}+f_{\text{ba}}^{\text{a}}-f_{\text{a}}^{\text{ba}}$$

(3) $${\left(\phi_{\text{bu}}\right)}_{t}+\nabla \cdot \left(u_{\text{b}}\phi_{\text{bu}}\right)=f_{\text{u}}^{\text{bu}}-f_{\text{bu}}^{\text{u}}-f_{\text{bu}}^{\text{ba}}$$

(4) $${\left(\phi_{\text{ba}}\right)}_{t}+\nabla \cdot \left(u_{\text{b}}\phi_{\text{ba}}\right)=f_{\text{a}}^{\text{ba}}+f_{\text{bu}}^{\text{ba}}-f_{\text{ba}}^{\text{a}}$$

(5) $${\left(c\right)}_{t}+\nabla \cdot \left(u_{\text{f}}c\right)=\nabla \cdot \left(D_{\text{c}}\nabla c\right)+C_{1}\left(f_{\text{u}}^{\text{a}}+f_{\text{bu}}^{\text{ba}}\right)-C_{2}c$$

The individual mobile platelets are regarded as having a negligible effect on the fluid motion; they and the chemical advect with the velocity **u**_f_ of the bulk flow as indicated in Eqs. (1), (2), and (5). As indicated in Eqs. (3)–(4), the bound platelets move at a different velocity **u**_b_. The equations for the two velocity fields, **u**_f_ and **u**_b_ are presented below. The mobile platelets and activating chemical move by diffusion with respective diffusion coefficients *D* and *D*_c_. The platelets’ “diffusivity” is mainly a result of local flow disturbances generated by red blood cell interactions [1].

Platelets can transition between populations and the rate at which platelets transition from platelet population *ϕ*_i_ to *ϕ*_j_ is denoted $f_{\text{i}}^{\text{j}}$. The functions $f_{\text{u}}^{\text{a}}$ and $f_{\text{bu}}^{\text{ba}}$ are the respective rates of *activation* for mobile and bound platelets; $f_{\text{u}}^{\text{bu}}$ and $f_{\text{a}}^{\text{ba}}$ are the respective rates of *binding* for mobile unactivated and mobile activated platelets; and $f_{\text{bu}}^{\text{u}}$ and $f_{\text{ba}}^{\text{a}}$ are the respective *unbinding* rates for bound unactivated and activated platelets, that is, the rates at which platelets detach from an aggregate. We describe these transition rates in more detail below. As indicated in Eq. (5), ADP is released at a rate proportional to the rate of platelet activation $f_{\text{u}}^{\text{a}}+f_{\text{bu}}^{\text{ba}}$ and its concentration decays at the rate *C*_2_*c*. We assume that platelets can be activated when the ADP concentration is above a prescribed threshold value *c*_T_, and, consequently, the rate of activation of mobile platelets is $f_{\text{u}}^{\text{a}}=R_{0}H\left(c-c_{\text{T}}\right)\phi_{\text{u}}$, where *R*_0_ is a constant, and *H*(·) is a (slightly smoothed) Heaviside step function. Bound unactivated platelets can also be activated by a second mechanism which is intended as a surrogate for force-dependent activation signals transmitted to these platelets through the GPIb*α*-vWF bonds that bind them to the thrombus [25–27]. Thus, bound unactivated platelets are activated at rate

(6) $$f_{\text{bu}}^{\text{ba}}=\left(R_{0}H\left(c-c_{\text{T}}\right)+R_{1}\right)\phi_{\text{bu}},$$

where $R_{1}=\frac{2}{5}R_{0}$.

#### 2.1.2 Coupled Movement of Bulk Blood Fluid and Growing Thrombus

The model treats the bulk blood as a viscous Newtonian fluid and treats the thrombus, made up of bound platelets, as a viscoelastic fluid. Both fluids can be present at each point in space; the composition of the mixture is described by the volume fractions of fluid *θ*_f_ and bound platelets *θ*_b_ which are related by *θ*_f_ + *θ*_b_ = 1. The two-fluid mixture moves according to two coupled sets of momentum equations Eqs. (7)–(8) and a “co-incompressibility” condition Eq. (9):

(7) $$\rho \left({\left(\theta_{\text{f}}u_{\text{f}}\right)}_{t}+\nabla \cdot \left(\theta_{\text{f}}u_{\text{f}}u_{\text{f}}\right)\right)=-\theta_{\text{f}}\nabla p+\nabla \cdot \left(\theta_{\text{f}}{\underset{=}{\sigma }}_{\text{fv}}\right)+C_{3}\frac{{\theta_{\text{b}}}^{2}}{{\left(1-\theta_{\text{b}}\right)}^{3}}\left(u_{\text{b}}-u_{\text{f}}\right).$$

(8) $$\rho \left({\left(\theta_{\text{b}}u_{\text{b}}\right)}_{t}+\nabla \cdot \left(\theta_{\text{b}}u_{\text{b}}u_{\text{b}}\right)\right)=-\theta_{\text{b}}\nabla p+\nabla \cdot \left(\theta_{\text{b}}{\underset{=}{\sigma }}_{\text{bv}}\right)+\nabla \cdot \left(\theta_{\text{b}}{\underset{=}{\sigma }}_{\text{b}}\right)+C_{3}\frac{{\theta_{\text{b}}}^{2}}{{\left(1-\theta_{\text{b}}\right)}^{3}}\left(u_{\text{f}}-u_{\text{b}}\right).$$

(9) $$\nabla \cdot \left(\theta_{\text{f}}u_{\text{f}}+\theta_{\text{b}}u_{\text{b}}\right)=0.$$

In these equations, the volume fraction of bound platelets in the thrombus is *θ*_b_ = v_plt_(*ϕ*_bu_ + *ϕ*_ba_), where v_plt_ is the volume of an individual platelet and *θ*_f_ = 1 − *θ*_b_ is the volume fraction of the fluid. The quantity *p* is a Lagrange multiplier, which we refer to as pressure, whose role is to enforce the co-incompressibility condition Eq. (9).

The viscous stress tensors ${\underset{=}{\sigma }}_{\text{fv}}$ and ${\underset{=}{\sigma }}_{\text{bv}}$ for the bulk fluid and bound platelet material, respectively, are given by:

(10) $${\underset{=}{\sigma }}_{\text{fv}}=\mu_{\text{f}}\left(\nabla u_{\text{f}}+\nabla {u_{\text{f}}}^{T}\right)+\left(\lambda_{\text{f}}\nabla \cdot u_{\text{f}}\right)\underline{\underline{I}},\;{\underset{=}{\sigma }}_{\text{bv}}=\mu_{\text{b}}\left(\nabla u_{\text{b}}+\nabla {u_{\text{b}}}^{T}\right)+\left(\lambda_{\text{b}}\nabla \cdot u_{\text{b}}\right)\underline{\underline{I}},$$

in which $\underline{\underline{I}}$ is the identity tensor. For both fluids, we relate the respective viscosity coefficients by *λ* = −*μ* so that their bulk viscosities are zero. In addition to Eq. (9), the two velocity fields are coupled through interphase drag forces, represented by the final term in Eqs. (7) and (8). We use the Kozeny-Carman formula [28] to relate the drag to the bound platelet volume fraction *θ*_b_. Finally, the quantity ${\underset{=}{\sigma }}_{\text{b}}$ in Eq. (8) is the stress tensor due to bonds between bound platelets. The constitutive equation for this stress is discussed in the next section.

#### 2.1.3 Platelet Binding Kinetics

Cohesion between platelets is mediated by molecular bonds that form when specific proteins from the blood plasma bind to corresponding specific receptors on the surfaces of two platelets [1]. In our model, we consider two types of bonds. In one, the plasma protein von Willebrand factor (vWF) forms a bridge between GPIb*α* receptors on two platelets. We refer to this as a “GVG” bond for GPIb*α*-vWF-GPIb*α*. It is important that the formation of a GVG bond does *not* require that the platelets first be activated, and GVG binding kinetics are strongly influenced by the local hydrodynamic conditions. For example, vWF molecules display transitions between compact/globular and elongated conformations, depending on the magnitude of the local shear and elongational stresses. Elongation of vWF exposes many binding sites on it for the GPIb*α* receptors on platelets [9] and recent evidence suggests that the affinity with which a vWF molecule binds GPIb*α* depends on the tensile stress within the vWF molecule [23]. In the second type of bond, the plasma protein fibrinogen forms a bridge between integrin *α*_IIb_*β*_3_ receptors on two platelets’ surfaces; in this case, both platelets must already have been activated prior to bond formation. We refer to this type of bond as an “AFA” bond, for *α*_*IIb*_*β*_3_-fibrinogen-*α*_*IIb*_*β*_3_.

We denote by *z*_gvg_(**x**, t) and *z*_afa_(**x**, t) the number densities of platelet-platelet bonds mediated by vWF and fibrinogen, respectively; they give the number of bonds per unit volume at time t that connect bound platelets at location **x** to bound platelets elsewhere. When stretched, each bond produces a force. If the bonds were not able to break, the forces would correspond to the stress in an elastic solid, but because bond lifetimes are finite, the bonds overall give rise to viscoelastic stresses. We describe these stresses using the viscoelastic stress tensors ${\underline{\underline{\sigma }}}_{\text{gvg}}(\text{x},\text{t})$ and ${\underline{\underline{\sigma }}}_{\text{afa}}(\text{x},\text{t})$; the total viscoelastic stress that appears in the bound platelet momentum equation, Eq. (8), is ${\underline{\underline{\sigma }}}_{\text{b}}={\underline{\underline{\sigma }}}_{\text{gvg}}+{\underline{\underline{\sigma }}}_{\text{afa}}$. In [10], we derived evolution equations for the number density of bonds and the associated stress tensor from the underlying two-scale model, under the assumptions that these bonds move at the velocity **u**_b_ of the platelets to which they are attached, are formed at rate *α* and break at rate *β*, and that each bond acts as a linear spring with rest-length zero. Here, we assume that evolution equations of a similar form apply for the number densities and stress tensors for both types of platelet-platelet bonds that we consider in the model:

(11) $${\left({\underset{=\text{j}}{\sigma }}^{0}\right)}_{t}+\nabla \cdot \left(u_{\text{b}}{\underset{=}{\sigma }}_{\text{j}}^{0}\right)={\underset{=\text{j}}{\sigma }}^{0}\underline{\underline{\nabla u_{\text{b}}}}+{\left({\underset{=\text{j}}{\sigma }}^{0}\underline{\underline{\nabla u_{\text{b}}}}\right)}^{T}+C_{4}\alpha_{\text{j}}\underline{\underline{I}}-\beta_{\text{j}}{\underset{=}{\sigma }}_{\text{j}}^{0},\;{\left(z_{\text{j}}\right)}_{t}+\nabla \cdot \left(u_{\text{b}}z_{\text{j}}\right)=\alpha_{\text{j}}-\beta_{\text{j}}z_{\text{j}},$$

for j = gvg, afa. The superscript on ${\underline{\underline{\sigma }}}_{\text{j}}^{0}$ indicates that it is the stress tensor under the assumption that bonds have a rest length of zero. As we discussed in [10], the inclusion of platelet bonds with zero rest length in the two-fluid model is problematic. Because the co-incompressibility condition Eq. (9) enables a thrombus to contract by squeezing out fluid, zero rest-length bonds could lead to bound platelet motions that collapse a thrombus to a point. To avoid this, we modify the stress tensors to account (approximately) for the bonds having nonzero rest length [11]. This is described briefly below.

We turn next to defining the bond formation and breaking rate functions in Eqs. (11). Because some of these functions depend on the length of bonds, we first explain how we estimate a local average bond length at (**x**, *t*). We need to estimate this because bond length is a microscale quantity that we do not explicitly track in this model. In the two-scale model from which the current model is derived, the distribution of each type of bond is described by a function *E*(**x**, **y**, *t*) defined so that *∫*_**y**_ *E*(**x**, **y**, *t*)*d***y** is the concentration of bonds between platelets at **x** and platelets at nearby points a (scaled) distance |**y**| away [10, 13]. In terms of *E*, the bond stress tensor ${\underline{\underline{\sigma }}}^{0}$ and bond concentration *z* are defined by the relations

(12) $${\underset{=}{\sigma }}^{0}(x,t)=\frac{1}{2}\int_{y}E(x,y,t)S_{0}yy^{T}dy^{T}\text{ and }z(x,t)=\int_{y}E(x,y,t)dy\text{, }$$

where, as in Eq. (11), ${\underline{\underline{\sigma }}}^{0}$ is the stress tensor that would obtain if each bond behaved as a linear spring with rest length zero and stiffness coefficient *S*_0_. It follows from these formulas that

(13) $$<|y{|}^{2}>(x,t)=\frac{\int_{y}E(x,y,t)|y{|}^{2}dy}{\int_{y}E(x,y,t)dy}=\frac{2}{S_{0}}\frac{\text{Trace}\left({\underset{=}{\sigma }}^{0}(x,\text{t})\right)}{z(x,t)},$$

from which we estimate the average length of bonds between platelets at **x** and platelets elsewhere by

(14) $$<|y|>(\text{x},t)\approx {\left(<|y{|}^{2}>(\text{x},t)\right)}^{1/2}={\left(\frac{2}{S_{0}}\frac{\text{Trace}\left({\underline{\underline{\sigma }}}^{0}(\text{x},\text{t})\right)}{z(x,t)}\right)}^{1/2}.$$

We define < |**y**| >_afa_ and < |**y**| >_gvg_ from the pairs ${\underline{\underline{\sigma }}}_{\text{afa}}^{0}$, *z*_afa_ and ${\underline{\underline{\sigma }}}_{\text{gvg}}^{0}$, *z*_gvg_, respectively, and use them as described below.

For AFA bonds, we assume that the bonds form at rate *α*_afa_ given by

(15) $$\alpha_{\text{afa}}(\text{x},t)=K_{\text{afa}}^{\text{ab}}n_{\text{afa}}^{\text{max}}\phi_{\text{a}}\left(n_{\text{afa}}^{\text{max}}\phi_{\text{ba}}-2z_{\text{afa}}\right)+K_{\text{afa}}^{\text{bb}}{\left(n_{\text{afa}}^{\text{max}}\phi_{\text{ba}}-2z_{\text{afa}}\right)}^{2},$$

in which *ϕ*_a_, *ϕ*_ba_, and *z*_afa_ are evaluated at (**x**, *t*). In Eq. (15), $n_{\text{afa}}^{\text{max}}$ is the total number of activated *α*_IIb_*β*_3_ receptors on an activated platelet’s surface; $K_{\text{afa}}^{\text{ab}}$ and $K_{\text{afa}}^{\text{bb}}$ are second-order rate constants; and $n_{\text{afa}}^{\text{max}}\phi_{\text{ba}}-2z_{\text{afa}}$ is the number density of *unoccupied α*_IIb_*β*_3_ receptors on bound activated platelets at location **x** at time t. Eq. (15) reflects our assumption that the rate of formation of AFA bonds depends on the concentrations of unoccupied *α*_IIb_*β*_3_ receptors on activated platelets, but not explicitly on the concentration of fibrinogen in the blood plasma. This is reasonable because the plasma concentration of fibrinogen is high [8]. The two terms on the right-hand side of Eq. (15) describe, respectively, formation of bonds between activated unbound platelets and bound platelets in a thrombus, and between pairs of bound platelets in a thrombus. As written, the term $K_{\text{afa}}^{\text{ab}}n_{\text{afa}}^{\text{max}}\phi_{\text{a}}\left(n_{\text{afa}}^{\text{max}}\phi_{\text{ba}}-2z_{\text{afa}}\right)$ implies that activated platelets can bind to bound platelets only if the activated platelets are at the *same spatial location* as the bound platelets. Consequently, the binding of activated platelets to a thrombus would not increase the spatial extent of the thrombus. As we did in [10, 29], we replace the factor $\left(n_{\text{afa}}^{\text{max}}\phi_{\text{ba}}-2z_{\text{afa}}\right)$ by a binding affinity function *η*_afa_ so that activated platelets within a platelet’s diameter distance of the thrombus can bind to it. In effect, we calculate *η*_afa_(**x**, *t*) by convolving the function $\left(n_{\text{afa}}^{\text{max}}\phi_{\text{ba}}-2z_{\text{afa}}\right)$ with a smooth Gaussian-like function with characteristic width equal to a platelet diameter.

We treat the formation of GVG bonds in a similar way, using the bond formation rate function

(16) $$\alpha_{\text{gvg}}=K_{\text{gvg}}^{\text{ab}}\left(n_{\text{gvg}}^{\text{max}}\left(\phi_{\text{a}}+\phi_{\text{u}}\right)\left(n_{\text{gvg}}^{\text{max}}\left(\phi_{\text{ba}}+\phi_{\text{bu}}\right)-2z_{\text{gvg}}\right)\right)+K_{\text{gvg}}^{\text{bb}}{\left(n_{\text{gvg}}^{\text{max}}\left(\phi_{\text{ba}}+\phi_{\text{bu}}\right)-2z_{\text{gvg}}\right)}^{2},$$

where $n_{\text{gvg}}^{\text{max}}$ is the total number of GPIb*α* receptors on a platelet’s surface. Because GPIb*α* receptors on all platelets are constitutively able to bind vWF, the number densities of both unactivated and activated platelets appear in Eq. (16). As for AFA bonds, we replace the expression $\left(n_{\text{gvg}}^{\text{max}}\left(\phi_{\text{ba}}+\phi_{\text{bu}}\right)-2z_{\text{gvg}}\right)$ by the corresponding binding affinity function *η*_gvg_. In this paper, we do not explicitly track the vWF concentration or the state of vWF molecules. Instead, we define the rate coefficients $K_{\text{gvg}}^{\text{ab}}$ and $K_{\text{gvg}}^{\text{bb}}$ to reflect the force-dependent affinity of vWF for GPIb*α* receptors [23] and to reflect the greater availability of vWF binding sites on elongated vWF molecules at higher elongation rates [9].

(17) $$K_{\text{gvg}}^{\text{ab}}(\text{x},t)=K_{\text{gvg}}^{\text{ab},0}\frac{1}{1+\text{exp}\left((\Delta G-f(\text{x},t)\Delta x)/k_{B}T\right)}H_{\dot{\epsilon }}\left(\dot{\epsilon }(\text{x},t)-{\dot{\epsilon }}_{\text{crit}}\right),$$

(18) $$K_{\text{gvg}}^{\text{bb}}(\text{x},t)=K_{\text{gvg}}^{\text{bb},0}\frac{1}{1+\text{exp}\left((\Delta G-f(\text{x},t)\Delta x)/k_{B}T\right)}.$$

**Fig. 1:**
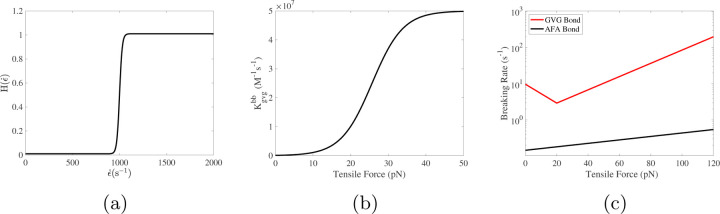
(a) Step-like function *H*_*ϵ*_. (b) Tension-dependent factor used in the vWF-GPIb*α* bond formation rate functions Eq. (17)–(18). (c) Dependence of AFA (*α*_IIb_*β*_3_-fibrinogen-*α*_IIb_*β*_3_) and GVG (GPIb*α*-vWF-GPIb*α*) bond breaking rates on the force on the bond. Note that the minimum rate of GVG bond breaking coincides with the beginning of rapid increase in the GVG bond formation rate.

In these formulas, $K_{\text{gvg}}^{\text{ab},0}$, $K_{\text{gvg}}^{\text{bb},0}$, Δ*G*, and Δ*x* are prescribed constants set to the average values measured by [23] (see Table 1), *f*(**x**, *t*) = *S*_gvg,0_(< |**y**| > (**x**, *t*) − *R*_gvg_) is the force sustained by a single GVG bond with rest length *R*_gvg_ when stretched to of length < |**y**| > (**x**, *t*); *k*_*B*_ is Boltzmann’s constant, *T* is absolute temperature. Also, $\dot{\epsilon }(\text{x},t)={\left\{{\left(\frac{\partial u_{f}}{\partial x}-\frac{\partial v_{f}}{\partial y}\right)}^{2}+{\left(\frac{\partial u_{f}}{\partial y}+\frac{\partial v_{f}}{\partial x}\right)}^{2}\right\}}^{1/2}$ is the fluid’s elongation rate at (**x**, *t*), ${\dot{\epsilon }}_{\text{crit}}$ is the critical elongation rate for vWF’s transition from a globular conformation to a stretched conformation, and $H_{\dot{\epsilon }}(\cdot )$ is a slightly smoothed version of the step function (shown in Fig. 1a) that increases from a basal level of 0.01 to 1.0 at ${\dot{\epsilon }}_{\text{crit}}$. See Fig. 1b for a plot of $K_{\text{gvg}}^{\text{bb}}$ as a function of *f*. Eqs. (17) and (18) differ because we assume that the vWF molecules that mediate further binding between pairs of already bound platelets are in the stretched conformation. In Eqs. (17)–(18), we use the average tension *S*_gvg,0_(< |**y**| > (**x**, *t*) − *R*_gvg_) in existing GVG bonds at (**x**, *t*) as a surrogate for the tension within a vWF molecule there.

The breaking rate of an individual AFA or GVG bond is known to depend on the force that bond is sustaining [5, 6], or equivalently on the length of the bond relative to its rest length. We use the quantities < |**y**| >_afa_ (**x**, *t*) and < |**y**| >_gvg_ (**x**, *t*) to approximate the bond lengths in the formulas for the breaking rates. For AFA bonds, the function has the form

(19) $$\beta_{\text{afa}}=\{\begin{array}{ll} \beta_{\text{afa}}^{\left(0\right)} & \text{ if }<|\text{y}|{>}_{\text{afa}}\leq R_{\text{afa}}\\ \beta_{\text{afa}}^{\left(0\right)}e^{\lambda_{\text{afa}}S_{\text{afa},0}\left(<|y|{>}_{\text{afa}}-R_{\text{afa}}\right)} & \text{ if }R_{\text{afa}}\leq <|\text{y}|{>}_{\text{afa.}} \end{array}$$

The bond stiffness *S*_afa,0_, the rest of length of the bond *R*_afa_, and the Bell’s law [24] parameters $\beta_{\text{afa}}^{\left(0\right)}$ and *λ*_afa_ > 0 can be estimated from existing experimental data [6]. The functional form of *β*_afa_ reflects our assumption that these bonds are “slip bonds” whose breaking rate is an increasing function of the force on the bonds. For GVG bonds, the form of the breaking rate function *β*_gvg_ involves two exponentials to reflect that these bonds act as “catch-slip” bonds for which small forces *reduce* the breaking rate and large forces increase it [5].

(20) $$\beta_{\text{gvg}}=\{\begin{array}{ll} \beta_{\text{gvg}}^{\left(0\right)} & \text{ if }<|\text{y}|{>}_{\text{gvg}}\leq R_{\text{gvg}}\\ \beta_{\text{gvg}}^{\left(0\right)}e^{\lambda_{\text{gvg}}^{\left(0\right)}S_{\text{gvg},0}\left(<|y|{>}_{\text{gvg}}-R_{\text{gvg}}\right)} & \text{ if }R_{\text{gvg}}\leq <|y|{>}_{\text{gvg}}\leq R_{\text{gvg}}^{CS}\\ \beta_{\text{gvg}}^{\left(1\right)}e^{\lambda_{\text{gvg}}^{\left(1\right)}S_{\text{gvg},0}\left(<|y|{>}_{\text{gvg}}-R_{\text{gvg}}\right)} & \text{ if }R_{\text{gvg}}^{CS}\leq <|y|{>}_{\text{gvg}}. \end{array}$$

Here, $\lambda_{\text{gvg}}^{\left(0\right)}<0$ and $\lambda_{\text{gvg}}^{\left(1\right)}<0$ and $R_{\text{gvg}}^{CS}$ is the bond length at which a transition from “catch” to “slip” behavior occurs. The breaking rate functions that we use for the two types of bonds are shown in Fig. 1c and Table 1 gives a complete list of model parameter values and their literature sources.

The rates of formation and breaking of platelet bonds are directly related to the rates of platelet attachment and detachment from an aggregate. Unactivated platelets can attach to a thrombus only through GVG bonds, while activated platelets can attach by both types of bonds. The transition rate function for initial attachment of an unactivated platelet is

(21) $$f_{\text{u}}^{\text{bu}}=\frac{1}{n_{\text{gvg}}^{\text{attach}}}K_{\text{gvg}}^{\text{ab}}n_{\text{gvg}}^{\text{max}}\phi_{\text{u}}\left(n_{\text{gvg}}^{\text{max}}\left(\phi_{\text{ba}}+\phi_{\text{bu}}\right)-2z_{\text{gvg}}\right).$$

and the transition rate function for initial attachment of an activated platelet is

(22) $$f_{\text{a}}^{\text{ba}}=\frac{1}{n_{\text{afa}}^{\text{attach}}}K_{\text{afa}}^{\text{ab}}n_{\text{afa}}^{\text{max}}\phi_{\text{a}}\left(n_{\text{afa}}^{\text{max}}\phi_{\text{ba}}-2z_{\text{afa}}\right)+\frac{1}{n_{\text{gvg}}^{\text{attach}}}K_{\text{gvg}}^{\text{ab}}n_{\text{gvg}}^{\text{max}}\phi_{\text{a}}\left(n_{\text{gvg}}^{\text{max}}\left(\phi_{\text{ba}}+\phi_{\text{bu}}\right)-2z_{\text{gvg}}\right),$$

In these equations, $n_{\text{afa }}^{\text{attach }}$ and $n_{\text{gvg }}^{\text{attach }}$ are the prescribed number of AFA and GVG bonds that we require be formed to achieve initial platelet attachment. The terms in Eqs. (21)–(22) correspond to similar terms in Eqs. (15)–(16) above, and, as discussed there, we replace $n_{\text{afa}}^{\text{max}}\phi_{\text{ba}}-2z_{\text{afa}}$ and $n_{\text{gvg}}^{\text{max}}\left(\phi_{\text{ba}}+\phi_{\text{bu}}\right)-2z_{\text{gvg}}$ by the corresponding binding affinity functions *η*_afa_ and *η*_gvg_, respectively.

The breaking of a bond results in a platelet detaching from the thrombus *only* if the bond broken is the last one connecting that platelet to the thrombus. Hence, the transition rates $f_{\text{bu}}^{\text{u}}$ and $f_{\text{ba}}^{\text{a}}$ at which bound, unactivated platelets and bound, activated platelets detach from the thrombus and become mobile are

(23) $$f_{\text{bu}}^{\text{u}}=\beta_{\text{gvg}}z_{\text{gvg}}P_{1}\frac{\phi_{\text{bu}}}{\phi_{\text{bu}}+\phi_{\text{ba}}},$$

(24) $$f_{\text{ba}}^{\text{a}}=\beta_{\text{gvg}}z_{\text{gvg}}P_{10}\frac{\phi_{\text{ba}}}{\phi_{\text{bu}}+\phi_{\text{ba}}}+\beta_{\text{afa}}z_{\text{afa}}P_{01},$$

respectively. In Eq. (23), *P*_1_ is the probability that an unactivated platelet has exactly one GVG bond. In Eq. (24), *P*_10_ is the probability that a platelet has exactly one GVG bond and no AFA bonds, and *P*_01_ is the probability that an activated platelet has no GVG bonds and exactly one AFA bond. These probabilities are calculated assuming that the numbers of bonds of each type for a specific platelet are distributed according to modified-Poisson distributions with averages $\frac{z_{\text{gvg}}}{\left(\phi_{\text{bu}}+\phi_{\text{ba}}\right)}$ and $\frac{z_{\text{afa}}}{\phi_{\text{ba}}}$ for GVG and AFA bonds, respectively. See the Supplement of [30] for details on the calculation of the probability functions. The functions $f_{\text{bu}}^{\text{u}}$ and $f_{\text{ba}}^{\text{a}}$ describe the rates of detachment of individual platelets from a thrombus. It also can happen that a mass of platelets embolizes (breaks away) from the thrombus. This can occur if the bonds holding this group of platelets to the rest of the thrombus break faster than they are replaced by new bonds. Embolization is not described by a prescribed function in the model, it is an emergent feature of the model.

The stress tensor ${\underline{\underline{\sigma }}}_{\text{j}}^{0}$, for *j* =afa, gvg, defined in Eq. (11) is appropriate if bonds between platelets are assumed to have zero rest length. The general definition of the stress tensor appropriate for rest length *R*_*j*_ is

(25) $${\underline{\underline{\sigma }}}_{j}(\text{x},t)=\frac{1}{2}\int_{y}E_{j}(x,y,t)S_{j,0}\left(1-\frac{R_{j}}{\left|y\right|}\right){\text{yy}}^{T}dy={\underline{\underline{\sigma }}}_{\text{j}}^{0}-{\underline{\underline{\sigma }}}_{j}^{\prime }$$

where ${\underline{\underline{\sigma }}}_{\text{j}}^{0}$ is defined in Eq. (11) and ${\underline{\underline{\sigma }}}_{j}^{\prime }$ is defined by

(26) $${\underline{\underline{\sigma }}}_{j}^{\prime }(\text{x},t)=\frac{1}{2}\int_{y}E_{j}(\text{x},\text{y},t)S_{j,0}\frac{R_{j}}{\left|y\right|}{\text{yy}}^{T}dy.$$

We are unable to express the integral defining ${\underline{\underline{\sigma }}}_{j}^{\prime }(\text{x},t)$ in terms of the current macroscale model’s variables and so we approximate it by its volumetric part $\frac{1}{3}\text{Trace}\left({\underline{\underline{\sigma }}}_{j}^{\prime }\right)\underline{\underline{I}}$. Further, we note that

(27) $$\begin{array}{l} \text{Trace}\left({\underline{\underline{\sigma }}}_{j}^{\prime }(\text{x},\text{t})\right)=\frac{1}{2}\int_{y}E_{j}(\text{x},\text{y},t)S_{j,0}R_{j}|\text{y}|d\text{y}\\ =\frac{1}{2}S_{0}^{j}R_{j}z_{\text{j}}(\text{x},t)\frac{\int_{\text{y}}E_{j}(\text{x},\text{y},t)|\text{y}|dy}{\int_{y}E_{j}(\text{x},\text{y},t)dy}\\ =\frac{1}{2}S_{j,0}R_{j}z_{\text{j}}(\text{x},t)<|y|>(\text{x},t)\\ \approx \frac{1}{2}S_{0}^{j}R_{j}z_{\text{j}}(\text{x},t)\sqrt{\frac{2}{S_{j,0}}\frac{\text{Trace}\left({\underline{\underline{\sigma }}}_{\text{j}}^{0}\right)(\text{x},\text{t})}{z_{\text{j}}(\text{x},t)}.} \end{array}$$

Using the last expression in Eq. (27), our approximation for ${\underline{\underline{\sigma }}}_{j}$ is

(28) $${\underline{\underline{\sigma }}}_{\text{j}}(\text{x},t)={\underline{\underline{\sigma }}}_{\text{j}}^{0}(\text{x},t)-\frac{1}{6}z_{\text{j}}(\text{x},t)S_{j,0}R_{j}\sqrt{\frac{2}{S_{j,0}}\frac{\text{Trace}\left({\underline{\underline{\sigma }}}_{\text{j}}^{0}\right)(\text{x},\text{t})}{z_{\text{j}}(\text{x},t)}\underline{\underline{I}}}.$$

Further discussion of this approximation can be found in [11].

## 3 Results

### 3.1 Problem Setup

**Fig. 2:**
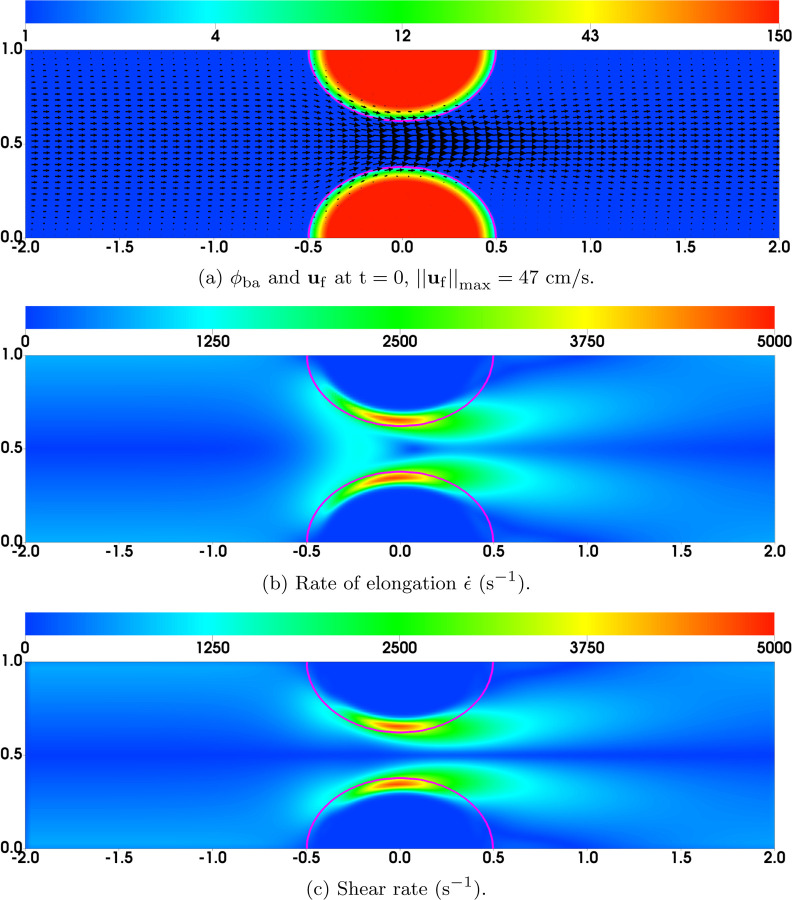
Steady state solution without platelet deposition used as initial conditions for simulations. The purple contour lines show the edge of the thrombi *ϕ*_ba_ = 2 at t = 0.

The computational domain is a 2D rectangular channel with a height of 1 mm and a length of 4 mm. A 256 × 64 uniform grid is used for all simulations. To mimic the stenotic flow channel used in experimental studies [11], two pieces of pre-formed dense thrombus are placed over the top and bottom walls of the domain. (This is more efficient than solving the PDEs in an irregular region whose geometry matches the stenosis.) As shown in Fig. 2a, the semi-elliptically shaped thrombi have a semi-major axis of approximately 0.5 mm and semi-minor axis of approximately 0.36 mm, so that, at its narrowest, only 28% of the lumen is open to unrestricted flow. At the initial time, the thrombi are composed of bound and activated platelets (number density *ϕ*_ba_) interconnected by AFA bonds. The value of *ϕ*_ba_ at t = 0 is set to 150*ϕ*_0_ in the cores of the pre-formed thrombi, where *ϕ*_0_ = 3 × 10^8^cm^−3^ is the platelet number density in the bulk blood. Near the edge of the thrombi, the profile of *ϕ*_ba_ transitions smoothly but sharply to zero. To maintain stability of the clots, the number density of AFA bonds is set to $z_{\text{afa}}=0.4n_{\text{afa}}^{\text{max}}\phi_{\text{ba}}$ so that each bound platelet has 2 × 10^4^ AFA bonds on average. The flow is driven by a constant pressure drop of 1.024 × 10^3^g · cm^−1^ · s^−2^ (1.024 × 10^2^ Pascal) over the length of the computational domain which gives a highest shear rate comparable to that measured in experiments [11]. For all simulations, both velocities **u**_f_ and **u**_b_ satisfy no-slip conditions at the top and bottom channel walls. Homogeneous Neumann conditions are applied to them at the inlet and outlet.

We begin with a pre-equilibration step to bring the fluid velocity and initial thrombi into steady state. To do this, we carry out simulations starting with **u**_f_ = **u**_b_ = 0 and reaching the approximate steady state flow plotted in Fig. 2 at about t = 4s. The highest fluid velocity is located at the center of the stenosis. The rate of elongation $\dot{\epsilon }$ for the flow is plotted in Fig. 2b. The region with the highest value for $\dot{\epsilon }\left(\approx 5000\;{\text{s}}^{-1}\right)$ is close to the edges of the thrombi where they are closest to one another. As shown in Fig. 2c, the shear rate distribution is very similar to that of the elongation rate. The steady state values of the model variables **u**_f_, **u**_b_, *ϕ*_ba_, *z*_gvg_, *z*_afa_, ${\underset{=}{\sigma }}_{\text{gvg}}$ and ${\underset{=}{\sigma }}_{\text{afa}}$ from the pre-equilibration step along with *ϕ*_u_ = 1 and *ϕ*_a_ = *ϕ*_bu_ = *c* = 0 are used as the initial conditions in all simulations presented below.

### 3.2 Effect of Bond Rupture on Thrombus Stability

**Fig. 3:**
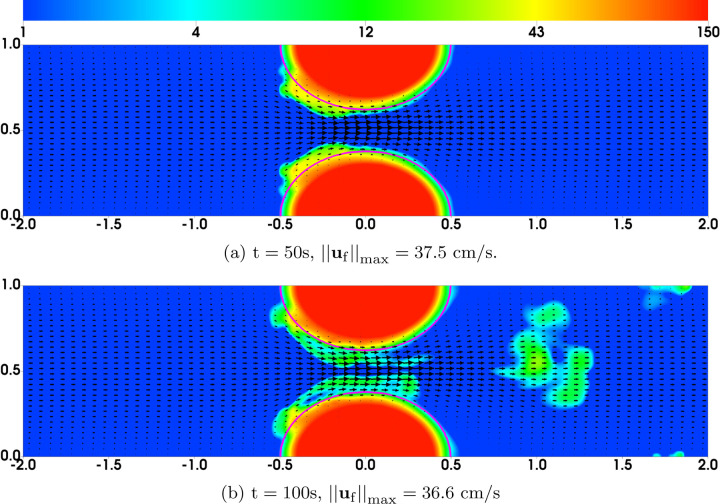
Bell Law Bond Rupture Rate Simulations: Distribution of *ϕ*_b_ = *ϕ*_bu_ + *ϕ*_ba_ and **u**_f_ from simulations with bond breaking rates Eqs. (19)–(20). Purple contours show the edges (*ϕ*_ba_ = 2) of the thrombi at t = 0.

In the first set of simulations (which we refer to as “Regular Bond Rupture”), we choose all model parameters as listed in Table 1. The time scale of platelet activation (by either the chemical agonist or mediated by GVG bonds) is set to *τ* = 5s, so *R*_0_ = 0.2/s. To match approximately the time scale for the formation of occlusive thrombi as observed in experiments [11], we choose the required numbers of bonds for initial platelet attachment, $n_{\text{afa}}^{\text{attach }}$ and $n_{\text{gvg}}^{\text{attach }}$ to be 20. The distribution of the bound platelet density (*ϕ*_ba_ + *ϕ*_bu_) and the fluid velocity **u**_f_ are plotted in Fig. 3 at t = 50s and t = 100s. At t = 50s, the clots exhibit some moderate growth beyond their initial profiles (indicated by the black contour lines), especially near the upstream edge. The growth causes an approximately 40% reduction in the flow rate relative to its initial value. By flow rate we mean *∫*_*y*_ *u*_*f*_(*y*)*dy* where integration is over a vessel cross-section. At t = 100s, large pieces of the thrombi embolize and are carried downstream by the flow. There is an about 45% reduction in flow rate at this time. Simulations continued for much longer times (up to 200s) show that the growing thrombi fail to fully occlude the channel due to repeated embolization. The greatest reduction in flow rate is no more than 55%.

In experimental studies using flow conditions similar to those in our simulations, occlusive thrombi are observed to form [11]. We therefore next investigated which of our specific model assumptions or parameter choices might contribute to the discrepancy between our results and those experiments. The mechanical strength of a thrombus is of critical importance for the thrombus’ stability under fast flow, and it depends on the formation and rupture of inter-platelet bonds. Indeed, a close look at out simulation results (presented later) indicates that embolization is mainly due to significant stretching and breaking of the GVG bonds in response to the fluid drag forces. According to our simulations, the average tensile force on individual GVG bonds can be as large as 200 pN. Most experimental studies to measure the rate of rupture for platelet bonds [5, 6] limit the magnitude of applied forces of 100 pN or less. Therefore, it is unclear whether the Bell’s law formulas for bond breaking given in (19) and (20) are valid for a tensile force as high as 200 pN.

**Fig. 4:**
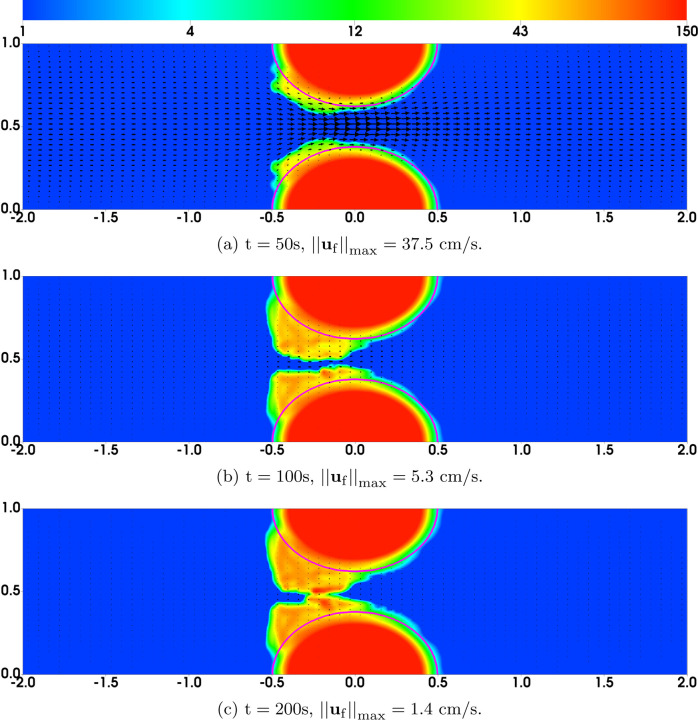
Plateauing Bond Rupture Rate Simulations: Distribution of *ϕ*_b_ = *ϕ*_bu_ + *ϕ*_ba_ and **u**_f_ from simulations with bond rupture rate that plateaus at tensile force 100 pN. The purple contours show the edge (*ϕ*_ba_ = 2) of the thrombi at t = 0.

**Fig. 5:**
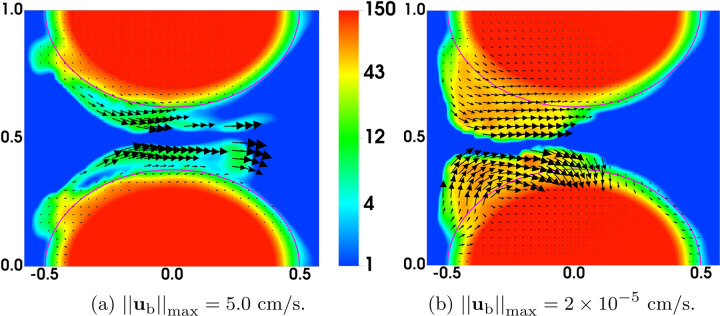
Comparison of Bond Rupture Rates: Distribution of *ϕ*_b_ and **u**_b_ from simulations with (a) regular and (b) plateauing bond rupture rate function at t = 100s. Note that the velocity vectors are scaled very differently.

As a simple strategy to explore these questions, we modified the bond rupture functions (19) and (20) in the second set of simulations (referred to as “Plateauing Bond Rupture”). We use those formulas to compute the rate of rupture for both GVG and AFA bonds only up to a tensile force of 100 pN and we use the rate for 100 pN also for all larger tensile force values. That is, we assume the breaking rates *plateau* for forces at and above 100 pN (corresponding to a bond stretched by 10 nM, 10% of its rest length). All other model parameters remain the same. The results are shown in Fig. 4. As seen from Figs. 4a–c, an “occlusive clot” is generated by time *t* = 200s, at which time the flow rate is reduced by approximately 97% relative to its initial value. The thrombus growth is mainly in the upstream region of the stenosis, where the flow is accelerating and the shear stresses are relatively high. The highest platelet number density in the thrombi over the stenotic region is about 120*ϕ*_0_, corresponding to a thrombus of porosity ≈ 0.64. Fig. 5 shows the bound platelet number density and velocity from the two simulations with regular and plateauing bond rupture rates. The thrombi generated in the simulation with plateauing bond rupture are much more stable than those from the simulation with the standard Bell’s law rupture rate.

**Fig. 6:**
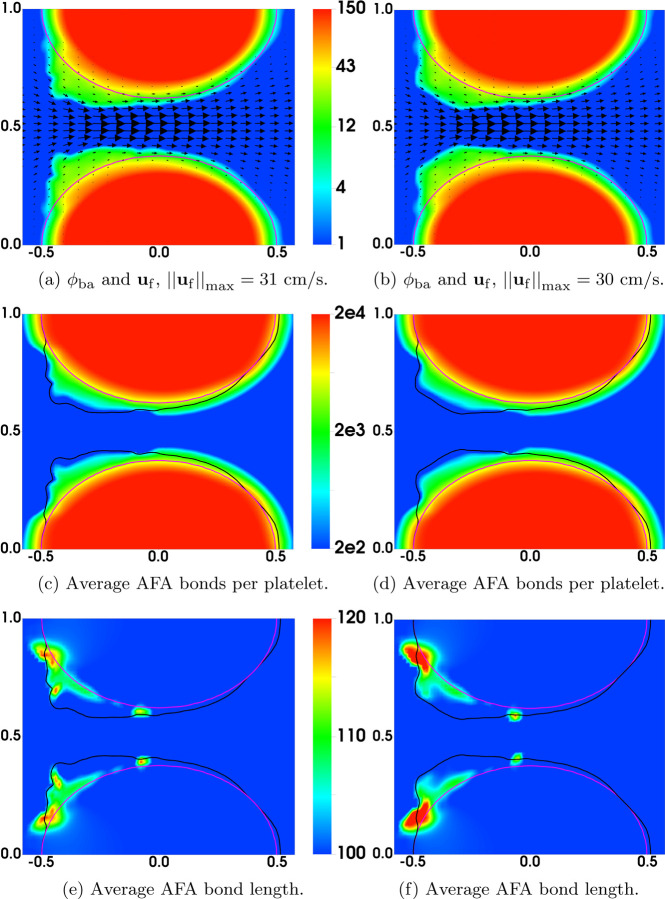
Comparison of Bond Rupture Rates: Simulations with (left) regular and (right) plateauing rupture rates at t = 60s. Contours *ϕ*_ba_ = 2 at (purple) t = 0 and (black) t = 60s.

**Fig. 7:**
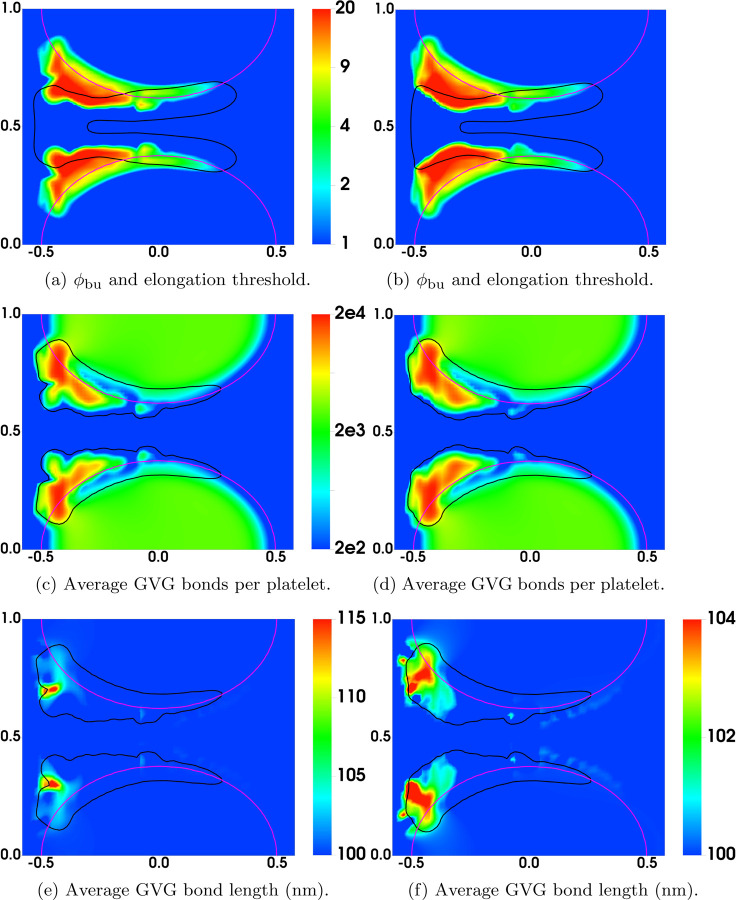
Results at t = 60s for simulations with (left) regular and (right) plateauing rupture rates. In (ab), the black contour lines show the elongation rate threshold (1000 s^−1^) for vWF stretching. The black contour lines in (c-f) show *ϕ*_bu_ = 2. The purple contours show the edges (*ϕ*_ba_ = 2) of the thrombi at t = 0.

Fig. 6 shows some model variables related to bound activated platelets at t = 60s, the time just before embolization first occurs. The left and right columns are from the simulations with regular and plateauing rates of rupture, respectively. As can be seen in Fig. 6ab, the distributions of *ϕ*_ba_ have similar profiles for the two simulations. As shown in Fig. 6cd, the average number of AFA bonds per deposited activated platelet (the region between the black and purple contour lines) varies from 200 to 2000 approximately. The average length of AFA bonds is shown in Fig. 6ef. The bonds are stretched up to 20% of their rest length (100 nm) near the upstream edges of the thrombi. Not surprisingly, the bonds are stretched more for the simulation with the plateauing rupture rate function. The distributions of *ϕ*_bu_ from the two simulations are shown in Fig. 7ab, where the black contour line indicates the assumed critical elongation rate $\dot{\epsilon }=1000\;{\text{s}}^{-1}$ for vWF molecules to transition from a globular to a stretched state. It is noticeable that for the simulation with regular rupture rate, the thrombi start to peel away along their upstream edges. Fig. 7cd show the average number of GVG bonds per bound platelet. This quantity reaches approximately 20,000 in the upstream portion of the thrombi where bonds are stretched the greatest amount. This is a consequence of the tension-dependent vWF activation (see Fig. 1b), suggested by the Springer lab [23], used in our model. The local average length of GVG bonds is shown in Fig. 7ef. At the time (*t*=60 s) of these plots, the GVG bonds are stretched up to 5 nm in the simulation with plateauing rupture rate. Notice that this corresponds to an average tensile force of 50 pN, which is large enough to activate high-affinity GPIb*α* -vWF binding (see Fig. 1b). For the simulation with regular rupture rate, the stretch length for the GVG bonds can be as high as 15 nm. The results in these snapshots may be surprising in that the average bond stretch for the plateauing case is substantially less than for the regular case, although long bonds break more rapidly in the regular case. At earlier times (not shown), average bond lengths in the plateauing case are much longer than those for the regular case in small spatial regions near the upstream end of the thrombi. The forces they generate keep that portion of the thrombus intact and allow more platelets to bind there and more bonds overall to form, and lead to a lesser force per bond at the time shown in Fig. 7

**Fig. 8:**
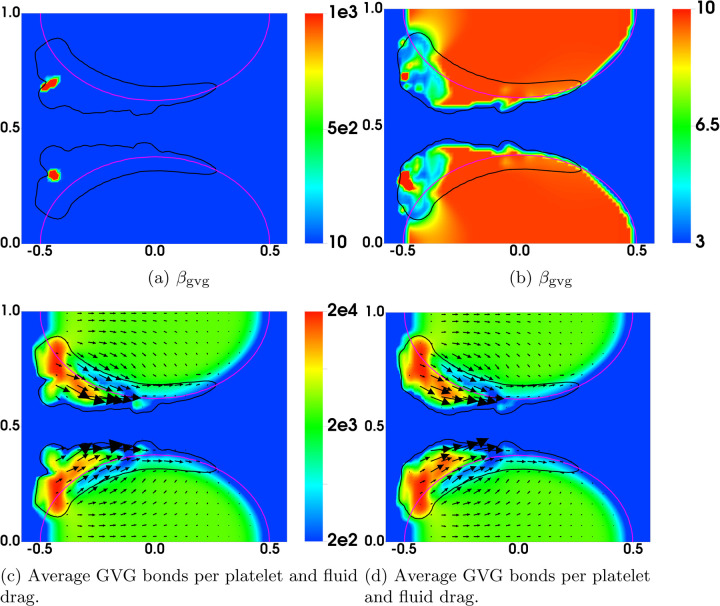
Results for simulations with (left) regular and (right) plateauing rupture rates at t = 60s. Black contours show *ϕ*_bu_ = 2. In (a-b), the net rate of breaking is scaled by $n_{\text{gvg}}^{\text{max}}\phi_{0}$. Note the different scales in (a) and (b).

Fig. 8ab display the rate of rupture for the GVG bonds at t = 60s. For the simulation with regular rupture rate, the largest value of *β*_gvg_ is around 1000s^−1^, which is two orders of magnitude higher than that for an unstretched bond $\left(\beta_{\text{gvg}}^{0}=10\;{\text{s}}^{-1}\right)$. The GVG bonds break most rapidly over highly localized regions of the thrombi, where small pieces of thrombi start to peel off. In comparison, in the simulation with plateauing rupture rate, GVG bonds break at a maximum rate (*β*_gvg_ = 12s^−1^) very close to $\beta_{\text{gvg}}^{0}$. For some regions of the thrombi near the upstream edge, the GVG bonds break with a rate much slower than $\beta_{\text{gvg}}^{0}$. Comparing this plot with Fig. 7f, we see that the GVG bonds over those regions are stretched about 2 nm (tensile force of 20 pN). Since the stretch is still within the catch-bond range (see Fig. 1c), the bond lifetime has actually increased with the applied tensile force. The distribution of the fluid drag force is shown in Fig. 8cd together with the average number of GVG bonds per platelet. For both simulations, the largest drag appears near the upstream edges of the thrombi.

**Fig. 9:**
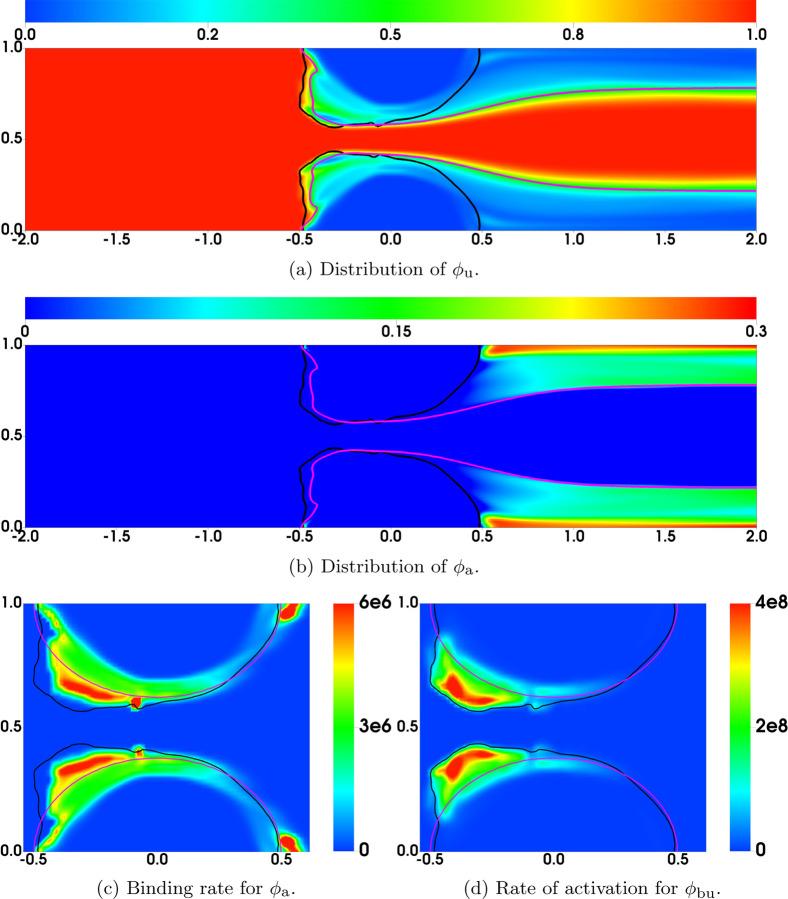
Results at t = 60s from a simulation with plateauing rupture rate. Black contours show *ϕ*_b_ = 2. Purple contours in a-b show the chemical concentration *c* = *c*_*T*_ required for platelet activation. Purple contours in c-d show the edges (*ϕ*_ba_ = 2) of the thrombi at t = 0.

In our model, platelets can be activated either by a chemical agonist, or as a consequence of the formation of GVG bonds, see Eq. (6). In Fig. 9ab, the distributions of *ϕ*_u_ and *ϕ*_a_ are plotted at t = 60s for the simulation with the plateauing bond rupture rate. In the region in which the agonist concentration is higher than the activation threshold (inside the purple contours), the density of unactivated platelets is relatively low because many platelets have already been activated. The highest value of *ϕ*_u_ in this region is near the upstream edges of the thrombi and is due to transport of unactivated platelets into these regions by the flow. The mobile activated platelet number density *ϕ*_a_ (Fig. 9b) is highest downstream of the thrombi including in the flow’s recirculation zones. Platelets become bound and activated in two ways, by the binding of activated fluid-phase platelets to the thrombus and by the activation of bound unactivated platelets. The rates at which these two processes occur are shown in Figs. 9cd, respectively. The fastest occurrence of both processes is near the upstream edges of the thrombi (although there is also significant binding of mobile activated platelets deep in the circulation zones downstream of the stenosis), and we see that the rate of binding of activated platelets in the fluid to the thrombus is negligible compared to the rate of activation of already bound platelets. From this observation, we conclude that binding of platelets to the thrombus through GVG bonds is almost a necessary precursor to the appearance of bound activated platelets from which AFA bonds can form.

### 3.3 Effect of Rate of Platelet Activation

In the previous section, the platelet activation time *τ* was set to a relatively short value of 5s. Thus, following the deposition of unactivated platelet through the formation of the relatively weak GVG bonds, platelets activation happens quickly and enables the formation of longer lasting AFA bonds that can help to maintain thrombus stability under fast flow. Note that real platelet activation is not an all-or-nothing process, the typical time scale for the activation of the platelets’ *α*_IIb_*β*_3_ receptors may be substantially different from other activation responses such as chemical secretion and the formation of pseudopodial appendages [35] and that rates of activation vary significantly among healthy persons [36]. In this section, we carry out simulations with a relatively long time scale for platelet activation. The goal is to investigate the importance of AFA bonds in maintaining thrombus stability under fast flow. We use the plateauing bond breaking rate for all simulations presented in this section.

**Fig. 10:**
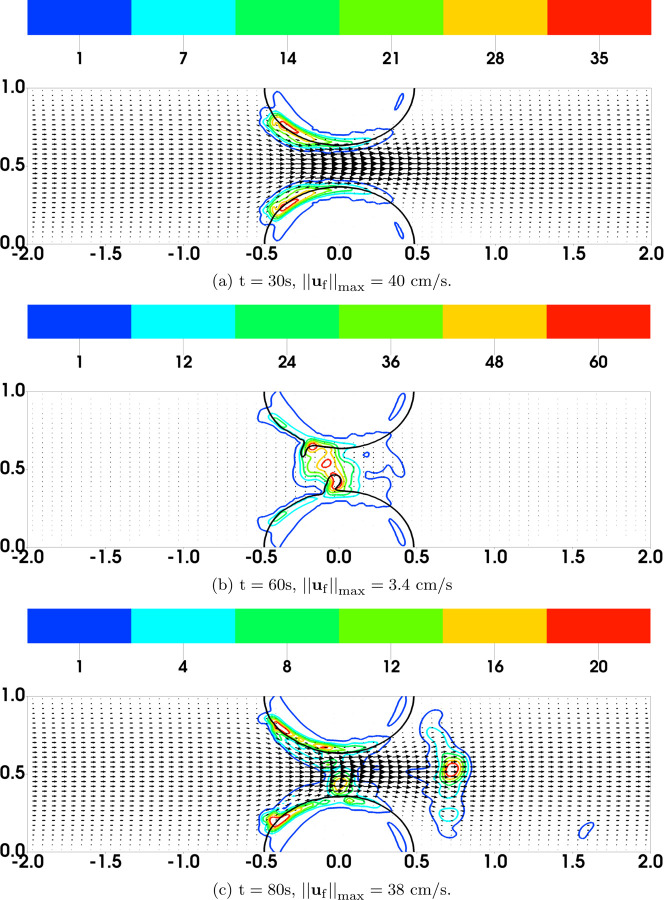
Simulation Results with Slow Platelet Activation. Distribution of bound platelets *ϕ*_b_ = *ϕ*_bu_ + *ϕ*_ba_ and fluid velocity **u**_f_. Black contours show *ϕ*_ba_ = 2. Note the different colorbar scales for *ϕ*_b_.

**Fig. 11:**
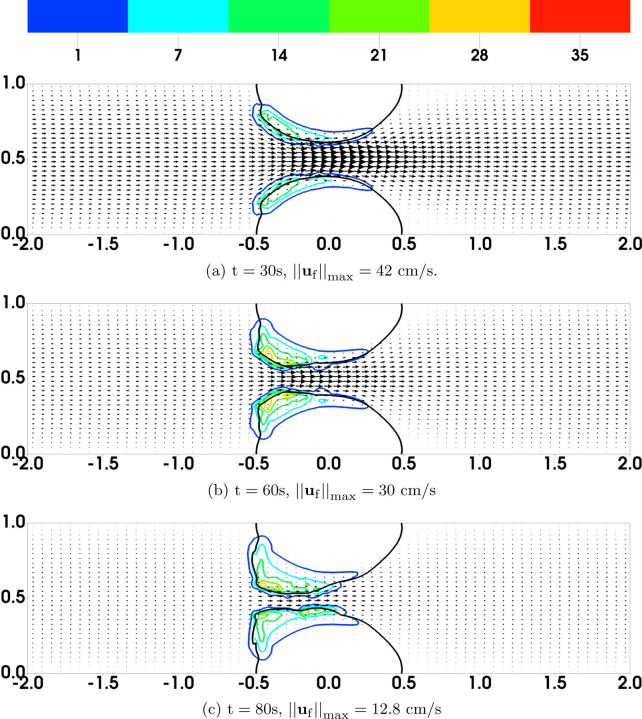
Simulation Results with Fast Platelet Activation. Distribution of bound platelets *ϕ*_b_ = *ϕ*_bu_ + *ϕ*_ba_ and fluid velocity **u**_f_. Black contours show *ϕ*_ba_ = 2.

**Fig. 12:**
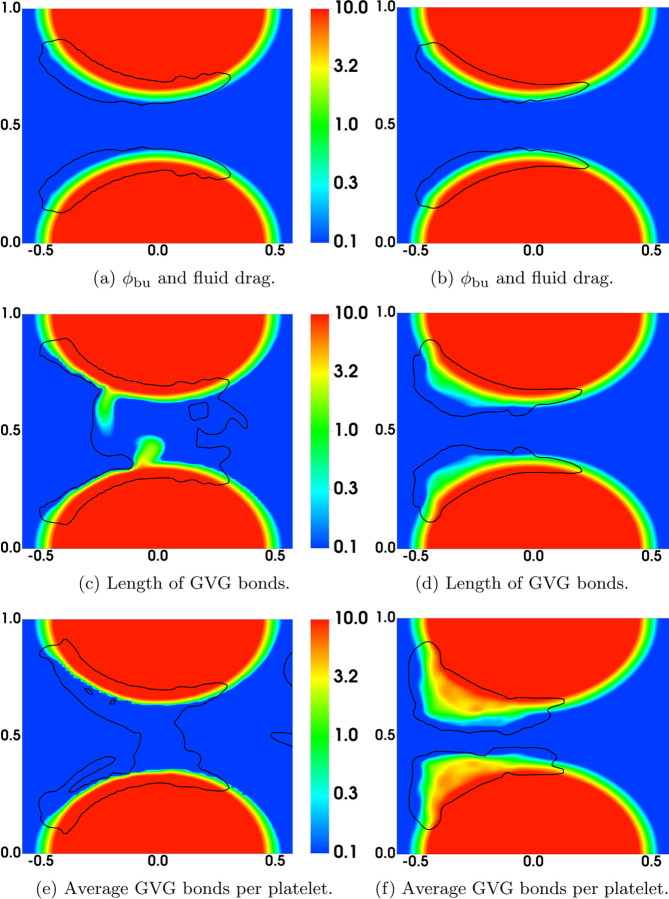
Distribution of various variables at t = 4.5s. (Left) $n_{\text{gvg}}^{\text{attach }}=n_{\text{afa}}^{\text{attach }}=100$. (Right) $n_{\text{gvg}}^{\text{attach }}=n_{\text{afa}}^{\text{attach }}=500$. Black contours show (a,b) *ϕ*_b_ = 2 at t = 0 and (c-f) *ϕ*_bu_ = 2 at t = 4.5s.

Figs. 10 and 11 show the distributions of bound platelets *ϕ*_b_ = *ϕ*_bu_ + *ϕ*_ba_ and the fluid velocity **u**_f_ from two simulations in which the time scale for platelet activation is *τ* = 40s and *τ* = 5s, respectively. The approximate edge of the region containing activated platelets (*ϕ*_ba_ ≥ 2) is shown by the black contours. In Fig. 10, we can compare the distribution of all bound platelets (colored contours) to that for bound activated ones (black contour) and infer that almost all of the deposited platelets are unactivated at t = 30s when the time scale for activation is *τ* = 40s. In fact, at this time, activated platelets are confined almost exclusively to where they are placed initially to represent the stenosis itself. Even at t = 60s, there are only small regions outside of this initial area in which *ϕ*_ba_ reaches a value of 2. With very low numbers of AFA bonds (see Fig. 12), the thrombi in this simulation become unstable at a later time. We see that large pieces of the thrombi have been peeled from the upstream end of the stenosis and carried downstream by the flow. At t = 60s (Fig. 10b), these emboli have become lodged in the narrow part of the stenosis, temporarily occluding the channel and causing a large drop in the maximum fluid velocity. However, the blockage is short-lived and by t = 80s (Fig. 10c), most of these platelets have been pushed further downstream, reopening the channel and allowing the maximum fluid velocity to become close to its initial value.

In contrast, Fig. 11a shows that, at t = 30s, there are many fewer bound unactivated platelets in the thrombi in the simulation with *τ* = 5s. By this time, the extent of the region in which there are bound activated platelets (*ϕ*_ba_ ≥ 2) has grown noticeably, and it continues to grow at later times. The thrombi remain stable (Fig. 11bc) with both unactivated and activated platelets distributed throughout the growing thrombi.

The distributions of AFA bond density (scaled by $n_{\text{afa}}^{\text{max}}\phi_{0}$) from the two simulations are shown in Fig. 12. When platelets are activated slowly (left column of the figure), few AFA bonds are found beyond their initial profile; they are absent from most of the region occupied by bound unactivated platelets. As a result, the thrombi, supported mainly by the relatively weak GVG bonds, are not stable under flow. By contrast, with much quicker platelet activation, large portions of the thrombi contain substantial numbers of activated platelets. This enables the formation of the stronger AFA bonds throughout the thrombi to stabilize them.

### 3.4 Effect of Rate of Platelet Bond Formation

In this final section, we look at an alternative way that stable occlusive thrombi might form. We revert to using the “regular” breaking rate functions Eq. (19)–(20) for all tensile force values, that is, without assuming that the rates have a plateau for tensile force greater than 100 pN. And we use a constant rate of GVG bond formation that is set to the maximum seen in [23], namely, $K_{\text{gvg}}^{\text{ab}}=5\times 10^{7}M^{-1}S^{-1}$. We note that this is much larger than the rate of formation of AFA bonds $K_{\text{afa}}^{\text{ab}}=1.6\times 10^{5}M^{-1}S^{-1}$. With these rates, thrombi form unrealistically rapidly (within a couple of seconds) if we continue to require that only $n_{\text{afa}}^{\text{attach }}=n_{\text{gvg}}^{\text{attach}}=20$ bonds are needed to mediate initial platelet attachment. Instead we set $n_{\text{afa}}^{\text{attach }}=n_{\text{gvg}}^{\text{attach}}=100$ or 500 in the simulations described in this section.

**Fig. 13:**
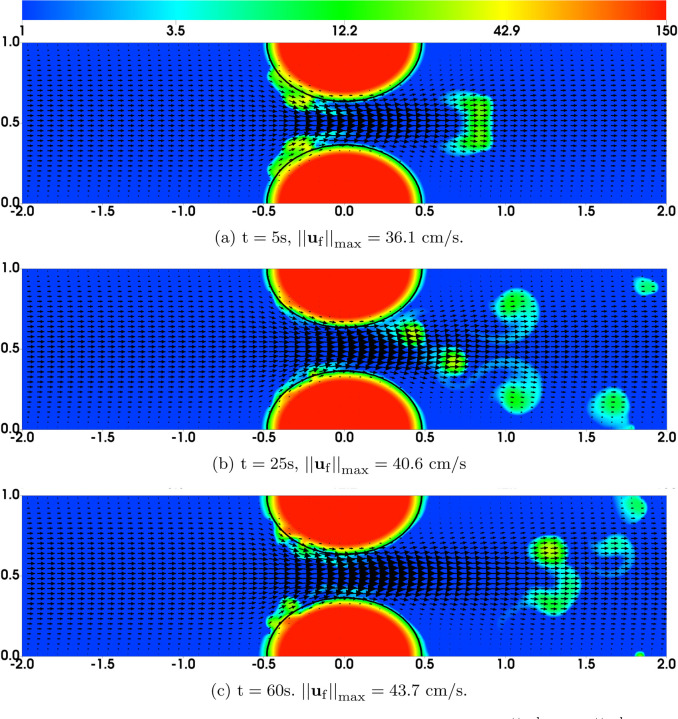
Distribution of *ϕ*_b_ and **u**_f_ at selected times with $n_{\text{gvg}}^{\text{attach }}=n_{\text{afa}}^{\text{attach }}=100$. Black contours show *ϕ*_ba_ = 2 at t = 0.

Fig. 13 shows the bound platelet number density *ϕ*_b_ = *ϕ*_bu_ + *ϕ*_ba_ and the fluid velocity **u**_f_ at selected times during a simulation with $n_{\text{afa}}^{\text{attach }}=n_{\text{gvg}}^{\text{attach}}=100$. With the high value of the rate constant for formation of GVG bonds in this simulation, platelets accumulate rapidly even with $n_{\text{gvg}}^{\text{attach}}=100$, much faster than seen in the previous sections. The rapid increase of *ϕ*_b_ causes a correspondingly rapid increase in the bound platelet volume fraction *θ*_b_ and of the magnitude of fluid drag in Eqs. (7) and (8). The rate of formation of AFA bonds is unchanged from the previous simulations, and so the rapid increase in drag is not matched by a rapid increase in *z*_afa_. As a consequence, the thrombi embolize. Continuation of the simulation to long time confirms that occlusive thrombi fail to form. Instead, we see the repeated growth and embolization of thrombi.

**Fig. 14:**
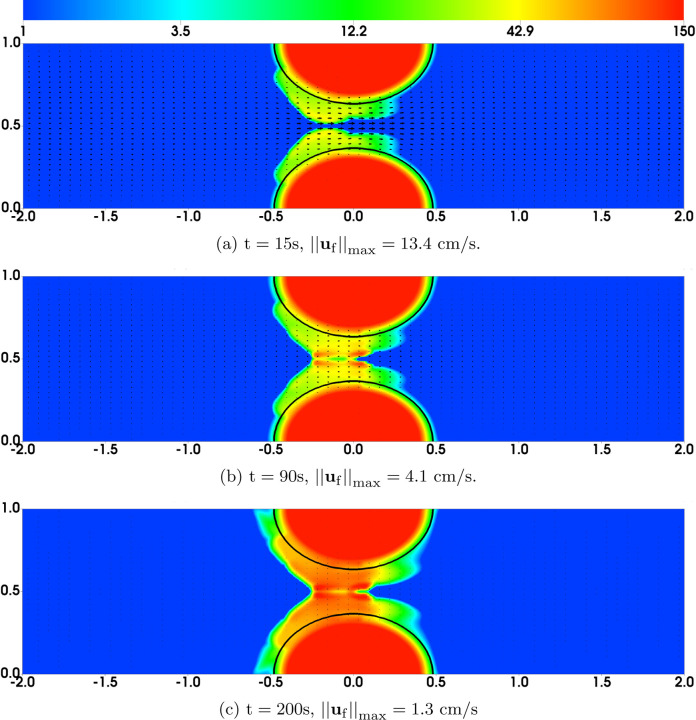
Distribution of *ϕ*_b_ and **u**_f_ at selected times for $n_{\text{gvg}}^{\text{attach }}=n_{\text{afa}}^{\text{attach }}=500$. Black contours show *ϕ*_ba_ = 2 at t = 0.

We performed another simulation with $n_{\text{afa}}^{\text{attach }}=n_{\text{gvg}}^{\text{attach}}=500$ and the same rate of GVG bond formation as in the simulation just described. Because only bound platelets contribute to a thrombus’s bound platelet volume fraction *θ*_b_, this change results in a more moderate rate of increase in the drag force on the thrombi and therefore bond formation has more chance to keep up with the increase in drag force. As shown in Fig. 14, the thrombi grow stably without any noticeable embolization. The channel is “fully occluded” by time 200s, at which time the flow rate is reduced by more than 98% from its initial value.

**Fig. 15:**
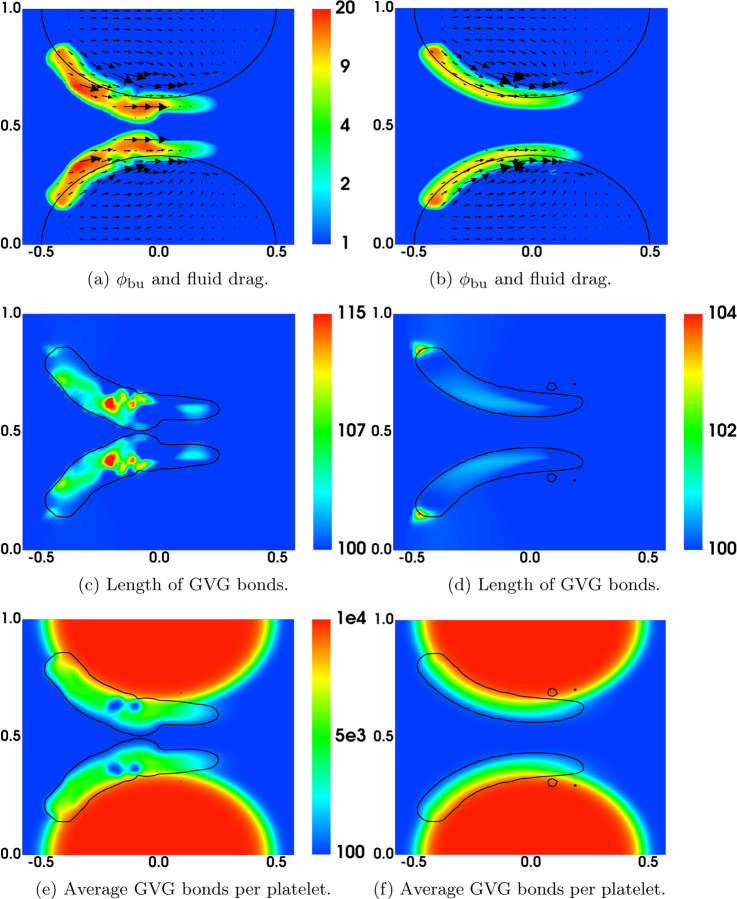
Distribution of various variables at t = 4.5s. (Left) $n_{\text{gvg}}^{\text{attach }}=n_{\text{afa}}^{\text{attach }}=100$. (Right) $n_{\text{gvg}}^{\text{attach }}=n_{\text{afa}}^{\text{attach }}=500$. Black contours show (a,b) *ϕ*_b_ = 2 at t = 0 and (c-f) *ϕ*_bu_ = 2 at t = 4.5s.

In Fig. 15, we compare the results at an early time (t = 4.5s) from the simulations with $n_{\text{afa}}^{\text{attach }}=n_{\text{gvg}}^{\text{attach}}=100$ and $n_{\text{afa}}^{\text{attach }}=n_{\text{gvg}}^{\text{attach}}=500$. From the plots of *ϕ*_bu_ in Fig. 15ab we see that the growth of the thrombus (i.e., the accumulation of bound platelets) is much greater with $n_{\text{afa}}^{\text{attach }}=n_{\text{gvg}}^{\text{attach}}=100$ than with $n_{\text{afa}}^{\text{attach }}=n_{\text{gvg}}^{\text{attach}}=500$. This, in turn, produces a much larger increase of drag force in the former case particularly in the regions where *ϕ*_bu_ is largest. The larger drag forces stretch the GVG bonds to a much greater extent (compare Fig. 15cd) and in the regions of greatest stretch, this leads to rapid bond breaking and a much lower number of bonds per platelet (Fig. 15ef). Note in particular the very low bond per platelet numbers in the blue regions within the thrombus in Fig. 15e at locations corresponding to the regions of highest drag and greatest bond stretching. For this simulation, embolization first occurs shortly after this time, and begins at these locations. For the simulation with $n_{\text{afa}}^{\text{attach }}=n_{\text{gvg}}^{\text{attach}}=500$, the moderate drag forces cause only slight elongation of the GVG bonds from their rest length. In much of the thrombus, the average bond length lies within the catch-bond length interval, and thus bond rupture occurs slowly. The average number of GVG bonds per platelet remains at a high level throughout the thrombi, contributing to their stable growth and allowing time for substantial AFA bond formation (not shown).

## 4 Discussion

In this paper, we investigated the formation of occlusive platelet thrombi within a stenotic channel in high shear flow. This is a critical process in arterial thrombosis in which the platelet aggregation dynamics is strongly affected by the local flow conditions. Our computational model is an extension of a previously developed two-phase platelet aggregation model [10, 11]. Specifically, a mechanism is included in the model so that the platelet deposition due to vWF-mediated bond formation is coupled with the elongation rate of the flow and the tensile force on existing platelet bonds [23]. The latter is used as a surrogate for the tensile force within vWF itself which the current model does not track. Our goal is to explore the connections between the formation of stable occlusive thrombi and several critical model parameters including the rate of bond rupture under large load, the platelet activation response times, and the rates of formation of interplatelet bonds. To accurately measure these in experiments is difficult and good data is lacking.

First, we investigated the effect that the rates of rupture for both platelet GPIba-vWF bonds and *α*_IIb_*β*_3_-fibrinogen bonds have on thrombus formation. We noticed that the experimental measurements of the rupture rates for these bonds with respect to the Bell Model were carried out under the tensile force ranging from 0–120 pN [5, 6]. So it is not clear if the Bell model and/or its parameters obtained from these experiments are pertinent for tensile forces of larger magnitude, that may arise under arterial flow conditions. We carried out two simulations that differ only in the functional form of the rupture rates for tensile forces greater than 100 pN.

For the simulation in which the rupture rates follow the Bell Model (termed “regular rupture rate”) for tensile forces of all magnitudes, the newly deposited platelet aggregates were repeatedly torn apart by the flow and occlusive thrombi failed to form. The results of this simulation indicate that at some locations within the thrombi the average force sustained by an individual bond has a magnitude as large as 200 pN. Thus, the GPIba-vWF bond, with its average life time as short as 10^−3^ s, cannot form sufficiently rapidly to compensate for the rapid bond breaking and to support stable thrombus growth.

For the other simulation, we assumed that the rupture rates reached their maximum at 100 pN and were constant for larger forces (we termed this the “plateauing rupture rate”). In this case, the thrombus growth was largely stable. Within minutes, the flow channel was completely “occluded” by the thrombi. A detailed comparison indicates that with the plateauing rupture rate, the average life time for the GPIba-vWF bond could be enhanced by two orders of magnitude relative to those in the simulation with the regular rupture rate. In early stages of the simulation, the stable thrombi are composed of both unactivated and activated platelets. We further observed that most of the activated platelets within the thrombi were there as a result of activation of unactivated platelets already attached to the thrombus by GPIba-vWF bonds. The results highlight the importance of vWF-mediated platelet aggregation/activation under high shear flow [1]. Based on the simulations, further investigation is warranted into the question of whether the Bell Model is suitable to describe platelet bond rupture under large tensile forces. In addition to the applicability of the Bell Model, other factors may also contribute to the observed results. For example, in addition to bound platelets, a thrombus also contains other components such as red blood cells and strands of vWF molecules. Thus it is possible that the mechanical stiffness of a bulk thrombus can be strengthened by these components, which are missing from our current model.

Next, we carried out a simulation with the plateauing rupture rate, but with the platelet activation time increased from 5 seconds to 40 seconds. We note that activation of real platelets involves multiple responses, including shape change, activation of integrin receptors, and secretion of soluble agonists, and that the time courses of the different responses are not well characterized. When the appearance of intraplatelet calcium spikes is used as an indication of initial activation, times range upwards from about 5 s [35, 37] and vary substantially among individuals [36]. In out simulation with the slower platelet activation rate, the stability of the thrombi was greatly reduced and stable occlusion did not happen. We observed transient occlusion, see Fig. 10, that was short-lived. According to the simulation, the initial fast deposition of platelets was mainly mediated by the fast formation of GPIba-vWF bonds. But since these bonds also break rapidly, the deposited platelets have to be activated quickly enough so that the thrombi can be stabilized under fast flow by the formation of stronger/longer lasting *α*_IIb_*β*_3_-fibrinogen bonds. Similar transient thrombus formation has been seen experimentally when fibrinogen-mediated binding is blocked [38], and in cases in which platelet activation is relatively slow [36].

We next performed simulations in which the rate of vWF-GPIb bond formation was fixed at the maximum rate 5(10)^7^*M*^−1^*S*^−1^ reported in [23] and consistent with other estimates that this rate is ≈ 100× the rate of *α*_IIb_*β*_3_-fibrinogen bond formation [2–5]. Our intent was to explore whether and under what circumstances this higher bond formation rate produces stable occlusive thrombi while using the standard Bell model for the bond breaking rate. In our first simulations with the fixed high vWF-GPIb on-rate and the “regular” Bell model off-rate, thrombi grew unrealistically rapidly but also very quickly embolized (results not shown).

This result drew our attention to the model parameter $n_{\text{gvg}}^{\text{attach}}$ in the expression (see Eq. (21)) for the rate $f_{\text{u}}^{\text{bu}}$ at which mobile unactivated platelets transition to being bound unactivated platelets. The value of $n_{\text{gvg}}^{\text{attach}}$ sets the number of vWF-GPIb*α* bonds that are required to mediate *initial* attachment of a mobile unactivated platelet to a thrombus. For these first new simulations, as well as those reported earlier in this paper, we used $n_{\text{gvg}}^{\text{attach}}=20$. Our reason for setting this parameter to a value greater than 1 is that it is well known that under high-shear conditions, the initial attachment of a platelet to a flow-chamber wall by vWF-GPIb*α* bonds generally does not bring that platelet to rest. Rather the platelet is seen to translocate or “roll” along the wall at a reduced speed, coming to a stop if sufficiently many bonds subsequently form or returning to the fluid if not [39–41]. Rough estimates for the required number of bonds range from only a few to several hundred [4]; the actual number likely depends strongly on how large the shear forces are. In the model, the consequence of a platelet transitioning from mobile to thrombus-bound is that the platelet then contributes to the local bound platelet volume fraction *θ*_b_ which enters into the calculation of the local drag force that appears in Eqs. (7)–(8) for **u**_f_ and **u**_b_. With a relatively low value of $n_{\text{gvg}}^{\text{attach}}$, a platelet’s contribution to the drag force begins when only a few bonds have formed between it and the thrombus. In reality, that platelet may not be firmly anchored to the thrombus, but may actually be rolling along its surface, and thus would contribute relatively little to the drag force.

Based on this reasoning, we carried out additional simulations with the high fixed vWF-GPIb*α* bond formation rate and the Bell model breaking, and using higher values of $n_{\text{gvg}}^{\text{attach}}$, i.e. $n_{\text{gvg}}^{\text{attach}}=100$ and $n_{\text{gvg}}^{\text{attach}}=500$. Even with $n_{\text{gvg}}^{\text{attach}}=100$, platelets accumulated much more rapidly than when using the tensile-force dependent vWF-GPIb*α* bond formation rate suggested by [23]. The rate of formation of the stronger AFA bonds was not able to keep up with the rapid increase in drag, and the thrombi were again seen to embolize and to not grow to occlude the vessel (Fig. 13). With $n_{\text{gvg}}^{\text{attach}}=500$, a balance was apparently achieved involving the growth in extent and density of the thrombi and the formation of bonds able to keep it intact. Stable thrombi grew to occlude the stenotic channel reducing the flow through it by 98%. (Fig. 14).

In summary, this paper shows that there is a strong interplay between the formation and breaking of both vWF-GPIb*α* and fibrinogen-*α*_IIb_*β*_3_ mediated platelet-platelet bonds, the rate of increase of a thrombus’ solid fraction (*θ*_b_) and the resultant reduction in the thrombus’ permeability, and the local flow in determining whether stable occlusive thrombi can form in a stenotic channel at high shear. Although our studies to date have been limited, some preliminary conclusions are that (i) there needs to be a balance between the rates of formation and breaking of vWF-GPIb*α* bonds in order to see a thrombus grow at a rate similar to that seen in experiments and to grow to form a stable occlusion of a stenotic channel, (ii) that it is critical to form sufficiently many vWF-GPIb*α* bonds early to initiate thrombus growth and later along the edges of developing thrombi, and (iii) formation of fibrinogen-*α*_IIb_*β*_3_ bonds within the core of a thrombus appears necessary for long-term thrombus stability, and therefore the rate of platelet activation by soluble agonists or by mechanical forces mediated by bonds places constraints on stable thrombus formation. Our conclusions are based on a model in which forces generated by platelet-platelet bonds are the sole means to resist the fluid dynamic forces on a growing thrombus. In actuality there may be other force sustaining structures, e.g., vWF bundles [42–44], that may share some of the load. We will explore this in future studies.

## Figures and Tables

**Table 1:** Values of model parameters.

Parameter	Definition	Value	Reference
*ρ*_f_ / *ρ*_b_	Density of fluid and bound platelets	1 g/cm^3^	-
*μ*_f_	Fluid viscosity	0.04 g/(cm ∙ s)	[1]
*μ*_b_	Bound platelet viscosity	100*μ*_f_	-
v_plt_	Volume of a single platelet	10^−11^cm^3^	[1]
*ϕ*_0_	Platelet number density in blood plasma	3 × 10^8^/cm^3^	[1]
*D*	Mobile platelet diffusion coefficient	10^−7^cm^2^/s	[31]
*C*_4_	Constant in stress equation (11)	$\frac{5}{3}\times 10^{-10}\;\text{g}\cdot {\text{cm}}^{2}/{\text{s}}^{2}$	[10]
*R*_*afa*_ = *R*_*gvg*_	Rest length of interplatelet bond	10^−5^cm	[32]
*S*_*afa*,0_ = *S*_*gvg*,0_	Stiffness of interplatelet bond	10pN/nm	[33]
$\beta_{\text{afa}}^{0}$	Breaking rate of unstretched α_IIb_*β*_3_-fibrinogen bond	0.06s^−1^	[6]
λ_afa_	Constant for strain dependent breaking rate in (19)	0.064/pN	[6]
$\beta_{\text{gvg}}^{0}$	Breaking rate of unstretched GPIbα-vWF bond	9.7s^−1^	[5]
*L*_*c*_	Critical length for catch-slip transition	102 nm	[5]
$\beta_{\text{gvg}}^{1}$	Constant for strain dependent breaking rate in (20)	1.256s^−1^	[5]
$\lambda_{\text{gvg}}^{1}$	Constant for strain dependent breaking rate in (20)	−0.06/pN	[5]
$\lambda_{\text{gvg}}^{2}$	Constant for strain dependent breaking rate in (20)	0.0422/pN	[5]
$K_{\text{afa}}^{\text{ab}}=K_{\text{afa}}^{\text{bb}}$	*α*_IIb_*β*_3_-fibrinogen bond formation rate	1.6 × 10^5^/(*M* ∙ *s*)	[34]
$K_{\text{gvg}}^{\text{ab},0}=K_{\text{gvg}}^{\text{bb},0}$	GPIb*α*-vWF bond formation rate	5.0 × 10^7^/(*M* ∙ *s*)	[23]
Δ*G*	Constant for strain dependent GPIb*α*-vWF bond formation rate in (17)(18)	6*k*_*B*_*T*	[23]
Δ*x*	Constant for strain dependent GPIb*α*-vWF bond formation rate in (17)(18)	1 nm	[23]
$n_{\text{afa}}^{\text{max}}$	Number of α_IIb_*β*_3_ receptors on a platelet surface	50,000	[1]
$n_{\text{gvg}}^{\text{max}}$	Number of GPIbα receptors on a platelet surface	25,000	[1]
